# Cyanoacrylate glue for iatrogenic retinal breaks during vitrectomy in stage 5 familial exudative vitreoretinopathy

**DOI:** 10.3389/fmed.2024.1370394

**Published:** 2024-06-20

**Authors:** Yihua Zou, Jie Peng, Peiquan Zhao

**Affiliations:** Department of Ophthalmology, Xinhua Hospital Affiliated to Shanghai Jiao Tong University School of Medicine, Shanghai, China

**Keywords:** cyanoacrylate, adhesives, retinal detachment, retinal breaks, familial exudative vitreoretinopathy, vitrectomy

## Abstract

**Purpose:**

To describe the role of cyanoacrylate glue in sealing iatrogenic retinal breaks (IRBs) during vitrectomy in stage 5 familial exudative vitreoretinopathy (FEVR) with funneled retinal detachment (RD).

**Methods:**

Nine eyes of nine patients diagnosed as stage 5 FEVR were treated with cyanoacrylate glue for IRBs during vitrectomy from July 2020 to January 2022. The clinical records, including patient information, surgical process, and follow-up examinations, were collected retrospectively. Anatomical outcomes and visual outcomes were summarized.

**Results:**

The average age at surgery was 19.6 months (range: 3.8–41.1 months). The mean post-operative follow-up period was 12.5 months (range: 9.8–18.8 months). Before surgery, five eyes had an open-funnel RD and four eyes had a closed-funnel RD. All the preretinal fibroplasia membranes were removed as thoroughly as possible in the nine eyes. IRBs formed at the posterior pole in two eyes and peripheral retina in seven eyes. All the IRBs were sealed successfully by the cyanoacrylate glue when they appeared. At the final post-operative visit, eight eyes had partial retinal reattachment without progression of fibroplasia tissues, while one eye had total retinal redetachment. The rate for stable anatomical outcome was 88.9% (8/9) in this study. The visual testing available for seven eyes demonstrated light perception in five eyes and no light perception in two eyes. No severe perioperative glue-related complications were noted during the follow-ups.

**Conclusion:**

The application of cyanoacrylate glue may be an alternative therapy for IRBs in stage 5 FEVR surgeries, while the long-term efficacy and safety still need further investigation.

## Introduction

Familial exudative vitreoretinopathy (FEVR) is one of the most common causes of irreversible blindness for pediatrics worldwide. It has been reported that FEVR that develops to various degrees of retinal detachment (RD) consists of about 21 ~ 85% of eyes (1–3). The pathogenic mechanism has been reported to be related with the traction of fibrovascular proliferation arising from abnormal neovascularization. Surgical intervention is helpful to prevent complications like glaucoma and phthisis bulbi but is limited to improve visual prognosis.

Surgery of stage 5 FEVR is one of the most troublesome situations of treatments for this disease. Iatrogenic retinal breaks (IRBs) can be a distressing issue during vitrectomy and predispose the surgery to fail if left untreated (4). The surgery is a challenge because of the particular characteristics: tight adhesion between the vitreous and retina, total retinal detachment, severe fibrovascular proliferation, and migration of pigment from the breaks into the vitreous cavity.

Cyanoacrylate adhesives have a strong strength of seal for retinal breaks in some vitreoretinal surgeries (e.g., choroidal coloboma, macular holes, and retinopathy of prematurity (ROP)) (5–7), whereas no relative study for advanced FEVR patients has been reported. The aim of this study was to describe the role of the cyanoacrylate glue in sealing the IRBs during vitrectomy for stage 5 FEVR patients with funneled RD.

## Patients and methods

All procedures were conducted in accordance with the Declaration of Helsinki and approved by the ethics committee of our hospital. Written informed consent for all surgical procedures was obtained from the legal guardians of the patients.

This study was a retrospective, interventional, single-surgeon, consecutive case series. Nine eyes of nine patients diagnosed as stage 5 FEVR were treated with cyanoacrylate glue for IRBs during vitrectomy from July 2020 to January 2022. The clinical records, including patient information, surgical process, and follow-up examinations, were collected retrospectively.

The diagnosis of FEVR was confirmed based on: (1) the full-term history, (2) peripheral avascular retina, macular dragging, or retinal folds, (3) positive genetic analysis, (4) similar fundus abnormalities of the parents/positive family history. The stage of FEVR was identified according to the classification scheme proposed by Pendergast and Trese (8). Stage 5 of FEVR was defined as total RD with open- or closed funnel configuration. All eyes had funneled RD with extensive preretinal fibrovascular proliferation beneath the posterior lens capsule. Eyes with obvious neovascularization, vitreous hemorrhage and questionable diagnosis (e.g., Norrie’s disease) were excluded.

**Figure 1:**
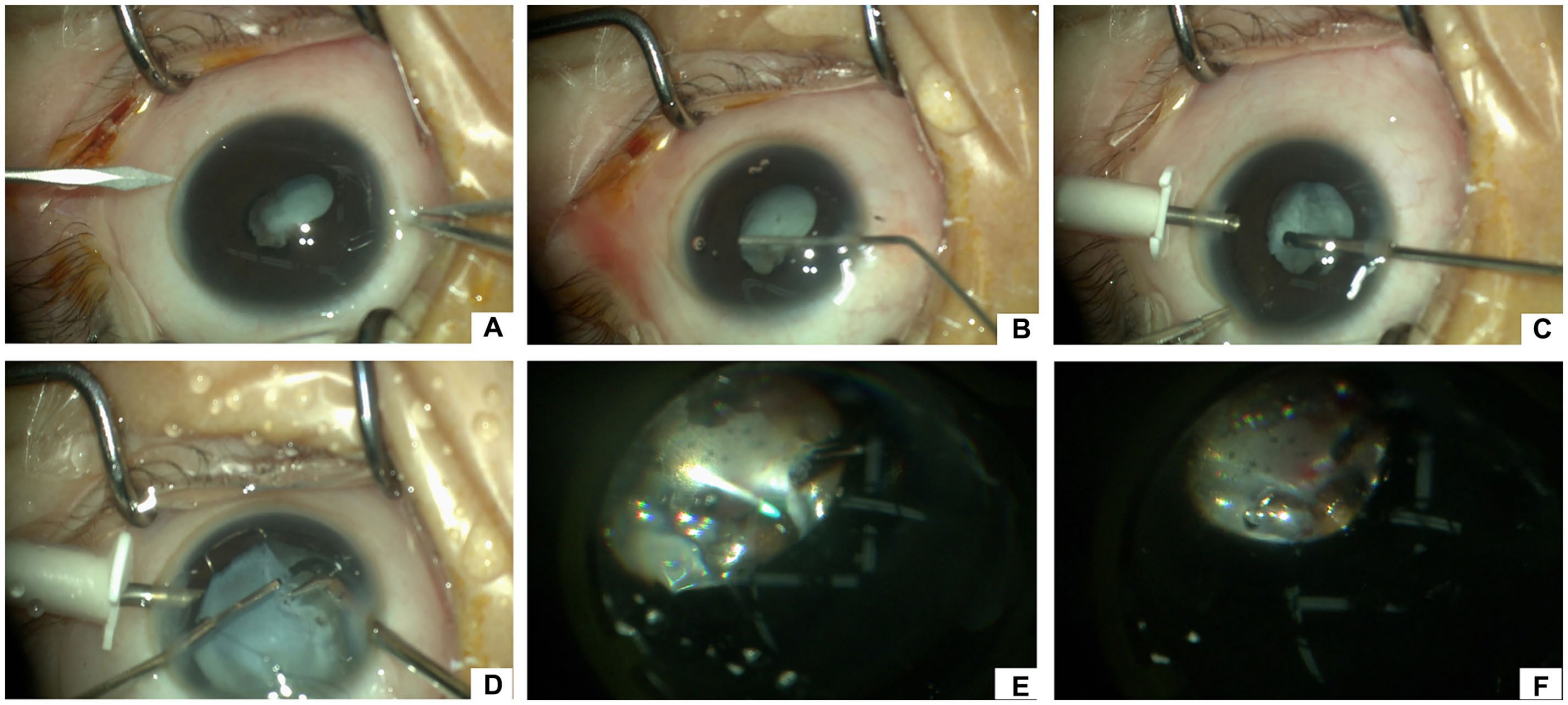
Surgical procedures of vitrectomy for stage 5 FEVR with iatrogenic retinal breaks. **(A)** Create a 20-G incision for the anterior chamber infusion line via the temporal limbal entry. **(B)** Inject viscoelastic into the anterior chamber to separate the anterior and posterior pupillary adhesion. **(C)** Perform 23-G lensectomy in phakic eyes for lens opacification or secondary glaucoma. **(D)** After injecting an iris speculum, perform the circumferential bimanual membrane dissection with 20-G end-gripping forceps and 20-G vertical scissors. **(E)** After trimming epiretinal fibrosis and condensed vitreous sheets by a 23-G vitreous cutter and fluid-air exchange, aspirate subretinal fluid through the iatrogenic retinal break(s) using a flute needle. **(F)** Inject one to several drops of cyanoacrylate glue to plug the retinal break(s) using a special injector. After that, fill the vitreous cavity and anterior chamber with viscoelastic, take out the iris speculum, and suture the limbal incisions.

All surgeries were performed by the same experienced surgeon. The process of surgery was according to the previous literature (9, 10) (Figure 1). A 20-G anterior chamber infusion line was created via the temporal limbal entry. Anterior and/or posterior pupillary adhesion was delaminated with viscoelastic. After synechiolysis, 23-G lensectomy was performed in phakic eyes for lens opacification or secondary glaucoma. An iris speculum was then injected via a superior 3.0-mm corneal incision to expand the pupil and expose the peripheral membrane. Iris hooks were used in one eye for better visualization. Circumferential bimanual membrane dissection proceeded toward the anterior retina with 20-G end-gripping forceps and 20-G vertical scissors. After the removal of retrolental membrane, residual epiretinal fibrosis and condensed vitreous sheets were trimmed by a 23-G vitreous cutter. After that, a fluid-air exchange was performed under coaxial illumination of the microscope. Subretinal fluid was aspirated through the IRBs using a flute needle.

**Figure 2:**
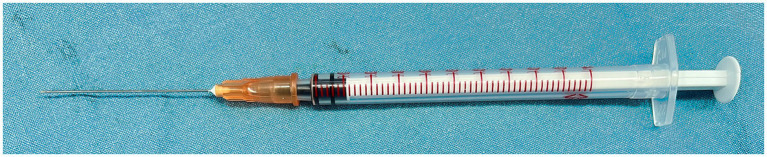
A special injector for the delivery of the glue. The injector is made up of a 25-G retrobulbar needle and a 1-mL syringe.

**Figure 3:**
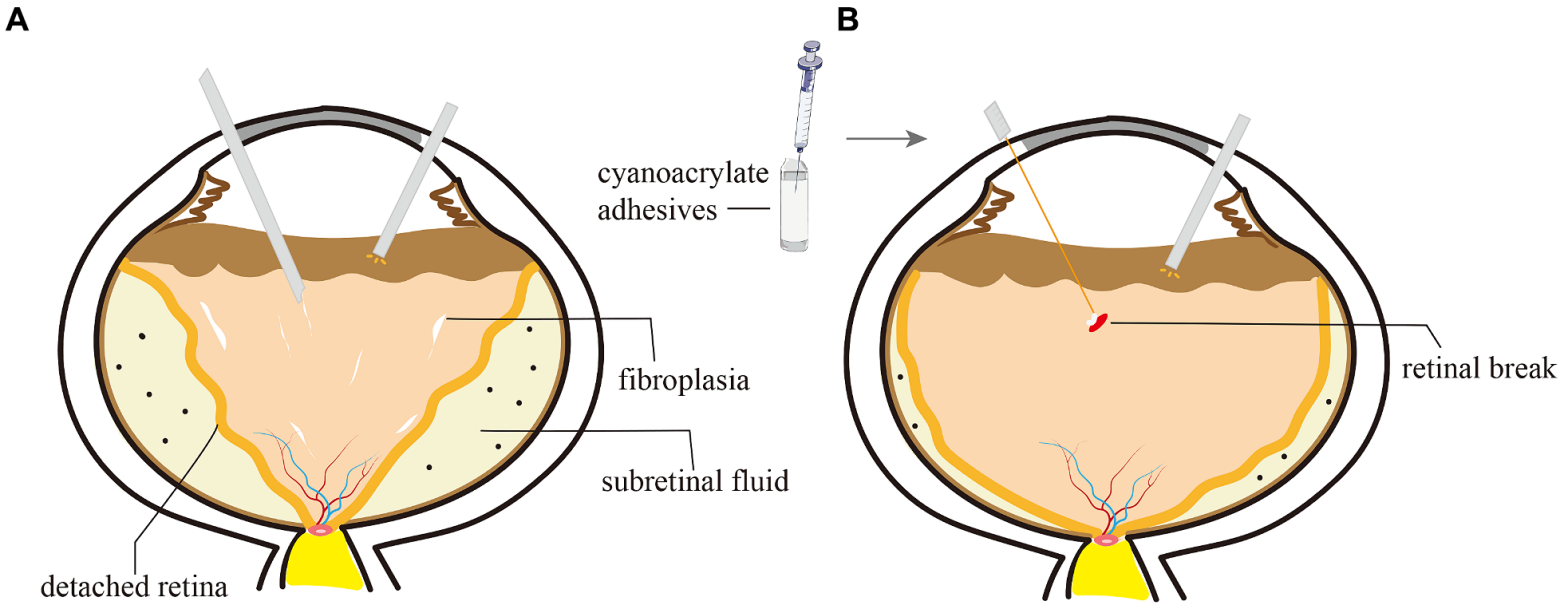
Schematic diagram of the surgical process of vitrectomy and the application of cyanoacrylate adhesives for iatrogenic retinal breaks (IRBs) in stage 5 FEVR. **(A)** Removing the vitreous and epiretinal fibroplasia by vitrectomy and microscope forceps. The severe and complex PVR changes make the detached retina become thick and stiff. **(B)** After drainage of subretinal fluid through the IRBs using a flute needle, the cyanoacrylate glue was injected and applied over the break using a special injector.

After thorough drainage of subretinal fluid, one to several drops of cyanoacrylate glue (FAL, Beijing Fuaile Medical Adhesive Co., Ltd., Beijing, China) were slowly injected to plug the IRBs using a special injector making up of a 25-G retrobulbar needle (outer diameter: 0.5 mm, inner diameter: 0.32 mm, length: 38 mm, TWLB L, KDL®, Zhejiang, China) and a 1-mL syringe (Figure 2). The syringe was quickly retracted from the vitreous cavity spared of perplexity with the glue. The glue formed a thin layer instantly within several seconds covering the retinal breaks. The vitreous cavity and anterior chamber were filled with viscoelastic to expand the retinal funnel and maintain intraocular pressure. The iris speculum was taken out from the anterior chamber with the injector. Limbal incisions were sutured with 10–0 nylon. The schematic diagram (Figure 3) and the Supplementary Video S1 (see video titled, “Cyanoacrylate Glue for Iatrogenic Retinal Breaks in Stage 5 FEVR”) demonstrates this technique.

**Figure 4:**
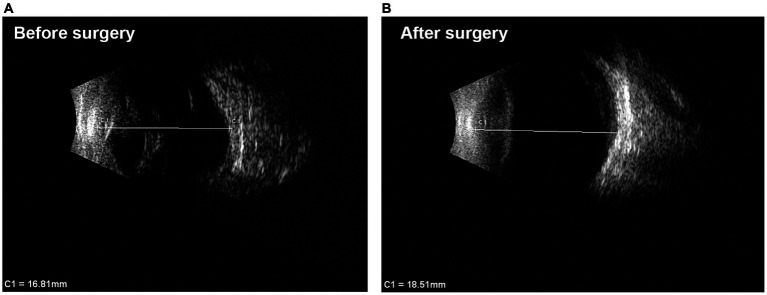
B-scan ultrasound images of one case before and after surgery. **(A)** Before surgery, the detached retina was a closed-closed configuration funnel. The axial length of the eye was 16.81 mm. **(B)** 14 months after surgery, the morphology of the eye became better and the axial length was 18.51 mm. The detached retina was open well although its thickness and stiffness make it difficult to become completely flat. Above all, the eye was saved and phthisis bulbi did not happen after the surgery.

The patients were evaluated on day 1, week 1, months 1 and 3, and every 3–6 months thereafter. At each visit, a complete eye examination was performed, including visual acuity, B-scan ultrasound (Figure 4), intraocular pressure measurement, anterior segmental examinations and color fundus photos by Retcam 3 (Clarity Medical Systems, Pleasanton, CA) or wide-field scanning laser ophthalmoscope (Optomap 200Tx, Dunfermline, United Kingdom). Patients were included in this consecutive series only if there was a minimum of 6 months of follow-up. Anatomical outcomes and visual outcomes were summarized.

## Results

The average age at the time of the surgery was 19.6 months (range 3.8–41.1 months). The mean follow-up period was 12.5 months (range 9.8–18.8 months). The male-to-female ratio was 8/1. Among the nine cases, family members of five cases were examined. A positive family history was identified in four cases, and the other case with a negative family history was identified by intraoperative observations. The fundus of the contralateral eyes in eight cases were diagnosed as FEVR, including five stage 5 FEVR, two stage 4B FEVR and two stage 1 FEVR. The fundus of the contralateral eye in one case was normal by fluorescein angiography examination. One case underwent gene testing and was identified with *NDP* gene mutation. The other three patients without family history or gene testing were diagnosed as FEVR by the features of a full-term history and retinal folds at the first visit or during surgeries.

Five eyes underwent primary vitrectomy and lensectomy due to a series of anterior segmental abnormalities (flat/shallow anterior chamber, iris synechia, cataract) and total RD (Table 1). Four eyes underwent initial lensectomy for secondary glaucoma. One eye (case 8) with abundant cholesterol crystals in the anterior chamber underwent secondary vitrectomy; the other three (case 2, 3, 6) underwent secondary vitrectomy for iris synechiolysis and removal of epiretinal fibroplasia (Table 1).

**Table 1:** Baseline information and follow-up outcomes of patients.

ID/ Sex/ Eye	Age at surgery (MO)	Anatomical outcome	Final VA	Follow-up time (MO)
1/M/OS	24.6	Partial reattachment	LP	10.1
2/M/OD	16.1	Partial reattachment	LP	9.8
3/M/OS	22.0	Partial reattachment	NLP	12.0
4/M/OD	13.8	Partial reattachment	LP	14.2
5/M/OD	41.1	Partial reattachment	LP	12.2
6/M/OS	16.4	Partial reattachment	NLP	18.8
7/M/OS	7.7	Partial reattachment	LP	12.9
8/M/OD	31.0	Redetachment	/	10.2
9/F/OS	3.8	Partial reattachment	/	12.2

MO, Month; VA, visual acuity; M, male; OD, oculus dextrus; OS, oculus sinister; F, female; LP, light perception; NLP, no light perception.

**Figure 5:**
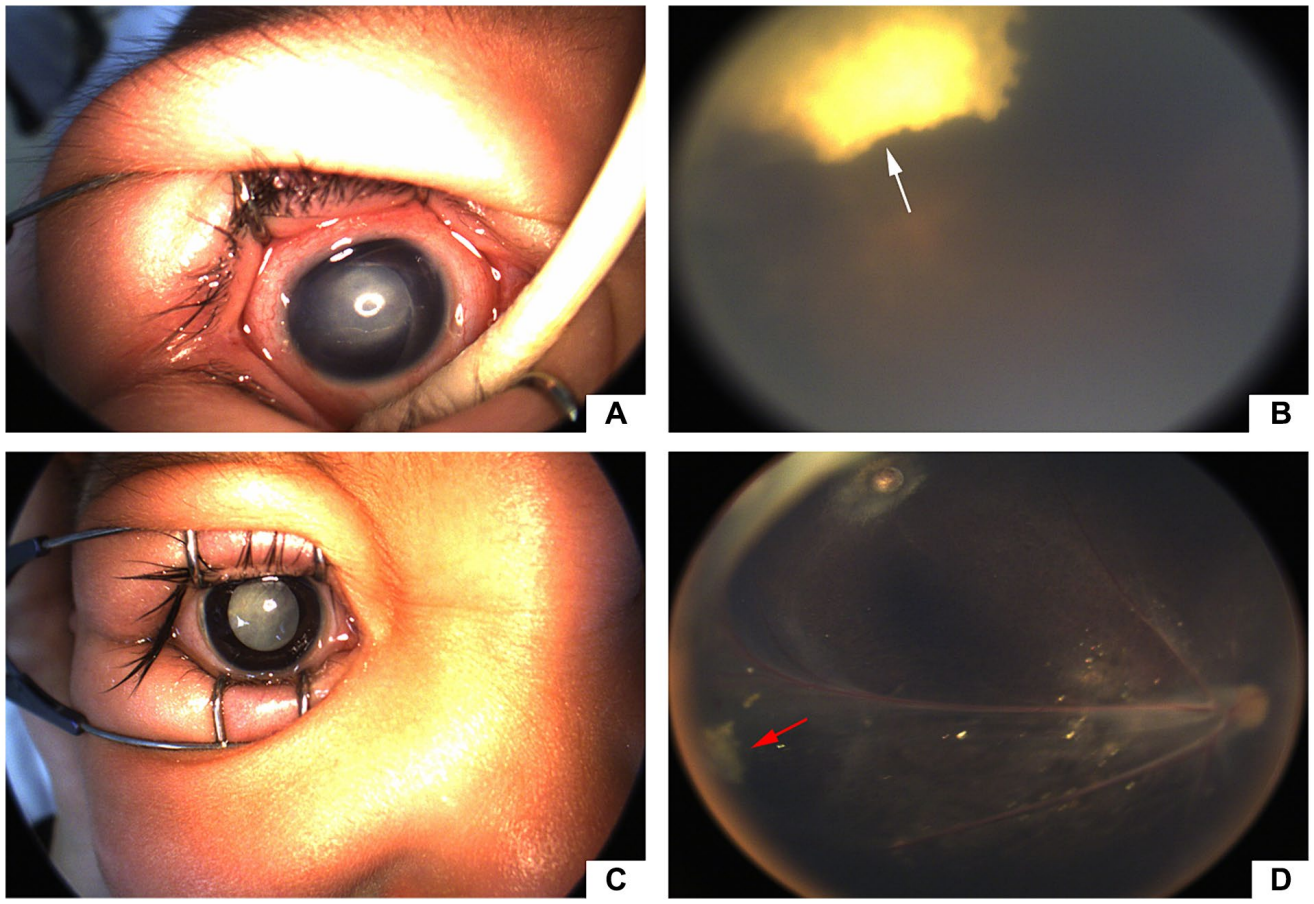
Preoperative and postoperative images of eyes. (a&b) Retcam images of a 16-month-old FEVR patient (case 2). **(A)** Before surgery, aphakic, abundant epiretinal fibroplasia and total RD. **(B)** 5 months after surgery, partial retinal reattachment and stable glue clot (white arrow) at the temporal periphery; (c&d) Retcam images of a 14-month-old FEVR patient (case 4). **(C)** Before surgery, abundant retrolental membrane and total retinal detachment (RD). **(D)** 14 months after surgery, nearly total retinal reattachment and stable glue clot (red arrow) at the temporal periphery.

After the removal of retrolental fibroplasia intraoperatively, five eyes had an open configuration funnel and four eyes had a narrow-closed or closed-closed configuration funnel. All the preretinal fibroplasia membranes were removed as thoroughly as possible in the nine eyes. IRBs formed at the posterior pole in two eyes and peripheral retina in seven eyes, including the temporal periphery in five eyes, the nasal periphery in one eye, and the superior periphery in one eye. The diameter of breaks was estimated to be from 1 mm to 3 mm. Until the final follow-up, eight eyes had partial retinal reattachment without progression of fibroplasia tissues, two of which had a flat retina at the posterior pole and macula (Figure 5), while one eye had total retinal redetachment. Therefore, the rate for stable anatomical outcome was 88.9% (8/9) in this study. The visual testing available for seven eyes demonstrated light perception in five eyes and no light perception in two eyes (Table 1).

During postoperative visits, one eye (case 1) had temporary elevated intraocular pressure which became normal after using topical carbonic anhydrase inhibitors; two eyes had a few vitreous cholesterol crystals. No complications related to the glue such as notable proliferative vitreoretinopathy (PVR), subretinal migration of the glue, iridocyclitis, vitreous hemorrhage, chorioretinitis, localized retinal edema and pigmentary changes were noted during the entire follow-up period.

## Discussion

The success rate of retinal reattachment for vitrectomy in FEVR patients ranged from 50 to 93% and visual improvement from 35 to 83% (11–14), varying significantly with the stage of FEVR. Vitrectomy for stage 5 FEVR is one of the most troublesome surgeries. The anatomical prognosis of this condition is relatively poorer. According to Fei et al. (10), vitrectomy for late-stage FEVR with severe complications achieved retinal reattachment in only 42% of eyes and partial reattachment in 58% of eyes.

IRBs can be a distressing issue during vitrectomy and predisposing surgical failure if left untreated. The vitreoretinal interface consists of a very adherent posterior hyaloid that cannot be easily peeled or perhaps peeled at all from the anterior retinal surface (15). Bimanual dissection by vitreous scissors and forceps can help remove the posterior hyaloid membrane but also have a high risk of IRBs. The retinal breaks provide a pathway for the migration of retinal pigment epithelial cells and subretinal fluid into the vitreous cavity, which further induces proliferative vitreoretinopathy and tractional retinal redetachment. On the other hand, common clinical treatments for retinal breaks (e.g., cryopexy, laser photocoagulation, scleral encircling) are not effective for stage 5 FEVR cases which have funneled retinal detachment and formation of strong retinal folds.

As there has not been efficient modalities for the above condition, the authors tried cyanoacrylate adhesives as an adjunctive technique during vitrectomy surgery. Cyanoacrylate adhesives are a kind of synthetic glue and can form a very strong seal and polymerize immediately on contact with fluid, thereby could be used as tissue glue. The advantages of cyanoacrylate adhesives included formation of an immediate, localized, and firm plug with lasting closure of the retinal break, which could facilitate retinal reattachment after relieving the retinal dragging by the fibroplasia tissues (16).

The cyanoacrylate glue used in this study is a commercial Chinese-made tissue adhesive, called Fuaile (FAL), which has been authorized to be applied in human surgeries in 2002 by the State Drug Administration (SDA) of China. FAL is composed of N-octyl-α-cyanoacrylate (NOCA; purity >99%) and N-butyl-cyanoacrylate (NBCA) (ratio 1:4). Both of the two cyanoacrylates have a relatively high molecular weight and less toxic than their lower molecular weight equivalents (methyl-cyanoacrylate) (16). This adhesive has a polymerization time ranging from two to six seconds and forms very stable and strong polymers in vivo (17). It has been widely used in various surgeries, such as gastrointestinal, gynecological, orthopedic, and neurosurgeries (18). For the first time, it was used in a small-scale case series of stage 5 FEVR surgeries in this study. The mean follow-up duration was 12.5 months. Eight eyes had partial retinal reattachment without progression of fibroplasia tissues at the final visit. The rate of stable anatomical outcome was not that high, which may be due to the relatively small number of cases. Besides, the nature of complicated and difficult surgical process for pediatric advanced FEVR may explain why the rate of complete retinal reattachment was relatively low.

Delivery of the glue in surgeries needs to be performed with great caution because of its rapid polymerization on contact with fluid. A specialized microinjector delivery system has been used in previous reports (19), but polymerization of the glue within the shaft of the microinjector could happen and need to be avoided. In this study, the special injector was made up of a 25-G retrobulbar needle and a 1-mL syringe, which was long enough for the delivery of the glue to the intraocular area of interest. The volume of the glue needs to be controlled with meticulous attention. Just one drop is often enough to form a thin layer of glue to seal the retinal break. Additional drops could be added according to the size of the retinal break.

The long-term safety of cyanoacrylate adhesives for the human retina has not yet been reported. The adhesives have been shown to have mild retinal histotoxicity (e.g., localized retinal edema, pigmentary changes) for created retinal breaks in experimental monkey and rabbit models (20, 21). In humans, short-term outcomes of cyanoacrylate adhesives in complicated retinal detachments, retinal breaks within choroidal coloboma, macular holes, and retinal breaks in advanced ROP have been observed, and no severe complications were identified (5–7, 22). Besides, the use of the adhesives was deemed essential to the success of the reported cases in these researches. In the five patients that Tyagi et al. (23) have reported, fibrin glue, a biological derived glue, along with laser photocoagulation could achieve closure of breaks in all with no tamponade and no postoperative positioning. However, fibrin glue has been observed to produce epiretinal proliferation. The possibility of the glue leading to PVR changes cannot be ruled out. Thus, the fibrin glue may be not suitable for stage 5 FEVR eyes with severe PVR changes. In this study, no obvious ocular complications, such as notable PVR, subretinal migration of the glue, iridocyclitis, vitreous hemorrhage, or chorioretinitis were noted in all eyes during the entire follow-up period. However, the long-term safety of the glue still needs further investigation in the future.

Other limitations of this study included the young age of cases and incomplete examinations. Due to noncooperation of the children, visual acuities of these patients are difficult to evaluate. However, a stable anatomical outcome could provide a possibility for residual low levels of vision, which has been proven to be helpful in infants and children, especially for those with bilaterally stage 5 FEVR. The safety and efficacy of the glue still need more large-scale and long-term prospective clinical studies to confirm.

In conclusion, this study explored the role of a cyanoacrylate glue in sealing IRBs during vitrectomy in stage 5 FEVR patients. The data of this pilot study indicate that this glue has advantages including fast polymerization and long-term closure of the break, which may be an alternative therapy for IRBs in stage 5 FEVR surgeries, while the long-term efficacy and safety of the glue still need to be further investigated.

## Data availability statement

The original contributions presented in the study are included in the article/Supplementary material, further inquiries can be directed to the corresponding authors.

## Ethics statement

The studies involving humans were approved by Ethics Committee of Xinhua Hospital, affiliated to Shanghai Jiao Tong University School of Medicine. The studies were conducted in accordance with the local legislation and institutional requirements. Written informed consent for participation in this study was provided by the participants' legal guardians/next of kin.

## Author contributions

YZ: Data curation, Formal analysis, Writing – original draft. JP: Funding acquisition, Methodology, Writing – review & editing. PZ: Funding acquisition, Methodology, Supervision, Writing – review & editing.

## Supplementary material

SUPPLEMENTARY VIDEO S1 Cyanoacrylate Glue for Iatrogenic Retinal Breaks in Stage 5 FEVR. After a fluid-air exchange and thorough drainage of subretinal fluid, one to several drops of cyanoacrylate glue (FAL, Beijing Fuaile Medical Adhesive Co., Ltd., Beijing, China) were slowly injected to plug the retinal break using a special injector. The syringe was quickly retracted from the vitreous cavity spared of perplexity with the glue. The glue formed a thin layer instantly within several seconds and covered the retinal breaks (red arrow).
